# Meta-analysis of the parasitic phase traits of *Haemonchus contortus* infection in sheep

**DOI:** 10.1186/s13071-017-2131-7

**Published:** 2017-04-24

**Authors:** Mathilde Saccareau, Guillaume Sallé, Christèle Robert-Granié, Tom Duchemin, Philippe Jacquiet, Alexandra Blanchard, Jacques Cabaret, Carole R. Moreno

**Affiliations:** 1 INRA - INPT-ENSAT - INPT-ENVT - Université de Toulouse, UMR 1388 GenPhySE, Castanet Tolosan, France; 2 INRA - Université de Tours, UMR 1282 ISP, 37380, Nouzilly, France; 3 INRA - INPT-ENVT - Université de Toulouse, UMR 1225 IHAP , Toulouse, France; 4 INRA - INPT-ENVT, UMT Santé des petits ruminants, Centre de recherche de Toulouse, Toulouse, France

**Keywords:** *Haemonchus contortus*, Gastro-intestinal nematode, Sheep, Meta-analysis, Resistance, Female fertility, Larval establishment, Adult mortality, Sex ratio

## Abstract

**Background:**

The parasitic nematode *Haemonchus contortus* shows highly variable life history traits. This highlights the need to have an average estimate and a quantification of the variation around it to calibrate epidemiological models.

**Methods:**

This paper aimed to quantify the main life history traits of *H. contortus* and to identify explanatory factors affecting these traits using a powerful method based on a systematic review and meta-analysis of current literature. The life history traits considered are: (i) the establishment rate of ingested larvae; (ii) the adult mortality rate; (iii) the fertility (i.e. the number of eggs laid/female/day); and (iv) fecundity of female worms (i.e. the number of eggs per gram of faeces).

**Results:**

A total of 37 papers that report single experimental infection with *H. contortus* in sheep and published from 1960 to 2015, were reviewed and collated in this meta-analysis. This encompassed 115 experiments on 982 animals. Each trait was analysed using a linear model weighted by its inverse variance. The average (± SE) larval establishment rate was 0.24 ± 0.02, which decreased as a function of the infection dose and host age. An average adult mortality rate of 0.021 ± 0.002) was estimated from the literature. This trait varied as a function of animal age, breed and protective response due to prior exposure to the parasite. Average female fertility was 1295.9 ± 280.4 eggs/female/day and decreased in resistant breeds and previously infected hosts. Average faecal egg count at necropsy was 908.5 ± 487.1 eggs per gram of faeces and varied as a function of infection duration and host resistance. The average sex ratio of *H. contortus* was 0.51 ± 0.006.

**Conclusion:**

This work is the first systematic review to summarise the available information on the parasitic phase of *H. contortus* in sheep*.* The results of the meta-analysis provide robust estimates of life history traits for parametrization of epidemiological models, their expected variation according to experimental factors, and provides correlations between these.

**Electronic supplementary material:**

The online version of this article (doi:10.1186/s13071-017-2131-7) contains supplementary material, which is available to authorized users.

## Background

The blood-feeding nematode *Haemonchus contortus* is one of the most pathogenic parasites in sheep [1]. The wide geographic distribution and increasing resistance against anthelmintic control measures has made this species a leading threat to the sustainability of sheep industries in Oceania, warm-humid regions [2] and Europe [3]. The critical impact on livestock production combined with the relative ease of producing parasite material in the laboratory have made this species a primary model of interest. For instance, specific genomic resources have been built to elucidate the genetic architecture of drug resistance mechanisms [4] and this species has also been used to evaluate alternative control strategies such as selective breeding for resistance to infection [5]. The success of these research avenues relies heavily upon the population dynamics and biology of *H. contortus* that both influence the diffusion of drug resistance alleles [6].

Mathematical models of parasite life-cycles provide an effective tool to advance epidemiological understanding [7, 8] and aid decision-making in parasite control strategies [9–12]. Indeed, the *in silico* approach allows for multiple scenarios to be considered without having to resort to experimentation once the model was validated [13–15]. These mechanistic models rely on the input of specific parameters, such as life history traits of the parasitic phase. Robust and average values for these traits are lacking, despite an extensive body of literature available on the subject. Recent systematic reviews and meta-analyses have been published to build knowledge of these parameters in other important gastrointestinal parasites such as *Cooperia oncophora* and *Ostertagia ostertagi* [16, 17] and *Teladorsagia circumcincta* [18]. Yet, despite the comprehensive literature available tackling the life-cycle of *H. contortus* [1], including both the free-living (eggs to infective larvae) and parasitic stages (infective larvae to reproductive adult worms), a quantitative assessment of *H. contortu*s population dynamics and ecology is still missing.

The aim of this paper was to conduct a systematic review and meta-analysis of data collected on experimentally infected sheep with *H. contortus,* published before 2016, using the same methodology as Verschave et al. [16]. The main life history traits selected for study in the parasitic phase of *H. contortus* included: (i) larval establishment; (ii) adult mortality; (iii) female fertility and population level fecundity. To obtain robust estimates of the life history traits, several experimental variation factors were considered i.e. infection dose, duration of the infection, host age, genetic background of host resistance and previous exposure to *H. contortus*.

## Methods

Verschave et al. [16] performed the systematic review and meta-analysis of *Ostertagia ostertagi* life traits in bovines. In this paper, the same approach was used for *Haemonchus contortus* life history traits. However papers reporting trickle infection results were removed as overlapping worm generations obscure worm age and thus prevent reliable estimation of larval establishment and adult mortality rates. Additional explanatory factors were studied, i.e. the influence of host resistance and of a previous exposure to *H. contortus* before the studied experiment. Some variance equations used in Verschave et al. [16] were corrected in this paper (Additional file 1: Text).

### Searching strategy and eligibility criteria

An exhaustive data collection was performed using the Web of Sciences database (https://www.webofknowledge.com) using the following keywords: ((gastrointestinal AND nematode) or (gastrointestinal AND parasite*) OR haemonchus OR contortus)) AND (infect* OR pathogen* OR mortality OR establishment OR fecundity) AND (sheep OR lamb* OR ewe* OR wether* OR ram* OR dam*). Selection of the papers used in the present study was completed on January 7^th^, 2016.

In the first review phase, papers were selected based on title compatibility. Papers without full text accessibility, unavailable through library services or published in languages other than French or English were discarded. Finally, the eligibility of the remaining papers was decided following a full reading of the text to meet the following criteria: (i) natural infections were excluded due to unknown parasite exposure; (ii) experiments which used anthelmintic treatments prior to sheep slaughter were not considered; and (iii) papers reporting their results graphically i.e. without precise values were removed.

In the second review phase, additional selection criteria were applied to the papers based on experimental design conditions. Insufficiently detailed experiments (i.e. the time between infection and slaughter) or nutritional supplementation studies (e.g. protein, zinc, selenium, papaya latex, condensed tannins) were withdrawn. Studies based on trickle infection protocols were also discarded, as larval stages from consequent infections will overlap with adult stages from the initial challenge making it impossible to correctly estimate larval establishment and adult mortality rates, particularly for infections of long duration. To ensure a robust meta-analysis, only experiments reporting the arithmetic means and associated measures of variance (standard deviation or standard error of the mean) for worm burden were kept for further analysis. Studies lacking experimental power (less than 3 animals) were ignored. Finally, the data were weighed according to their associated variances during estimation procedures. As atypical behaviour of variances were obtained for underpowered experiments (less than 10 animals), experiments showing too large or too small worm burden variance estimate (i.e. two standard deviations from the mean associated variance) were discarded.

### Parameter definition and specific eligibility criteria

The life history traits of *H. contortus* considered were: larval establishment which was estimated as the proportion of ingested larvae retrieved at necropsy, irrespective of the developmental stage (i.e. *L*_4_, immature stages, adult worms), the adult mortality rate (i.e. the proportion of adult worms that die per day), the female fertility and the population level fecundity. As defined by Southwood [19], fertility corresponds to the individual capacity of a female to lay eggs (measured by the total daily egg output in sheep faeces divided by the total number of female worms), whereas fecundity corresponds to the capacity of a population to lay eggs (measured by the total daily egg output in faeces). For each considered life history trait, the equations used to calculate mean estimates and their associated variances are summarised in Table 1 and detailed in Additional file 1: Text.

**Table 1:** Mean estimate and associated variance of the main life history traits of *H. contortus*

	Larval establishment (E)	Adult mortality (μ)	Female fertility (F)	Population level fecundity (f)
Definition	Proportion of ingested larvae that develop into immature or adult worms	Proportion of adult worms that die per day	Mean number of eggs laid in sheep faeces by an adult female per day	Mean number of eggs counted per gram of faeces
Mean estimate	$\frac{WB}{ID}$	$\frac{- ln\left(\frac{WB}{ID}\right)}{t}$	$\frac{ F E{ C}_n\ast DFP}{WB\ast {F}_p}$	*FEC*_*n*_
Variance	$\frac{SE{(WB)}^2}{I{ D}^2}$	$\frac{SE{(WB)}^2}{{\left( t\ast WB\right)}^2}$	$\frac{DF{ P}^2}{F_p^2}\ast \frac{ F E{C_n}^2}{W{ B}^2}\ast \left(\frac{SE{\left( FE{C}_n\right)}^2}{ F E{C_n}^2}+\frac{SE{(WB)}^2}{W{ B}^2}\right)$	*SE* (*FEC*_*n*_)

*Abbreviations*: *WB* Worm burden, *ID* Infection dose, *t* days after infection, *FEC*_*n*_ Faecal egg count at necropsy, *DFP* Daily faeces production, *F*_*p*_, proportion of female worms, SE Standard error

To disentangle larval establishment rate from adult mortality rate, we assumed according to expert knowledge that no adult worms die before 30 days post-infection (dpi), hence estimating larval establishment rate from experiments lasting less than 30 days and adult mortality rate from longer experiments. The worm’s sex ratio was estimated from experiments reporting separately female and male worm numbers. The number of female worms was assumed by this sex ratio estimation when unknown. Daily faeces production (DFP, g/day) was estimated as a direct function of host body weight (BW, kg) [20]:

$$ D F P\ \left( g/ day\right)=0.041\ast 1000\ast B{W}^{0.75} $$

Body weight that was missing in one paper [21] using Texel sheep was inferred from the paper of Freetly et al. [22] who derived a function linking body weight and age in Texel sheep.

The general study description (country, year, number of sheep), sheep-host details (breed, gender, age, body weight, previous exposure to *H. contortus*), *H. contortus* details (isolate, drug resistance status, infection dose) and experimental conditions (infection duration, concomitant infection with other nematode species) were included in our database. Host resistance to gastrointestinal nematodes was inferred from the literature, where Barbados Black Belly, Saint Croix, Florida Native [23], Santa Ines [24, 25], Canaria hair breed [26] and divergent selection lines were considered resistant breeds. Two *H. contortus* isolates were included: anthelmintic resistant (thiabendazole-resistant strain) or *H. contortus* isolates (ISE isolates) obtained after serial passages in resistant hosts.

### Meta-analysis

Statistical analyses were performed using R version 3.1.3. [27]. The life history trait estimates and their associated variances were calculated for each experiment of our final database (Table 1). Each life history trait estimate was analysed using a linear fixed model (*stats* package) weighted by the inverse of their associated variance. The considered explanatory variables were the host age (in months), the host resistance (i.e. susceptible, resistant, unknown status), the infection dose (i.e. the number of L_3_ inoculated per animal), the infection duration (i.e. number of days post-infection), previous exposure to *H. contortus* (i.e. immunologically naïve, natural pre-infection assumed, experimental pre-infection). Due to the skewed distribution of the life history traits, they were studied with, and without, a log transformation in further analyses. Quantitative explanatory variables (i.e. host age, infection dose and duration of infection) were clustered into balanced classes as the hypothesis of linearity between life history traits and explanatory variables was rejected.

First, a backward variable selection was implemented based on AIC criterion [28]. Then, only factors with a *P*-value < 0.05 were kept in the final models. *F*-tests were calculated to select significant factors using the *car* R package [29]. The estimation of means for each level of factors was performed using the *lsmeans* R package [30].

As each life history trait had a different inverse variance weight, it was impossible to use the same criteria to weight the correlation between life history traits. Consequently, correlations between life history traits were weighted by the number of sheep involved in the experiment using the ‘weight’ R package [31].

## Results

**Fig. 1:**
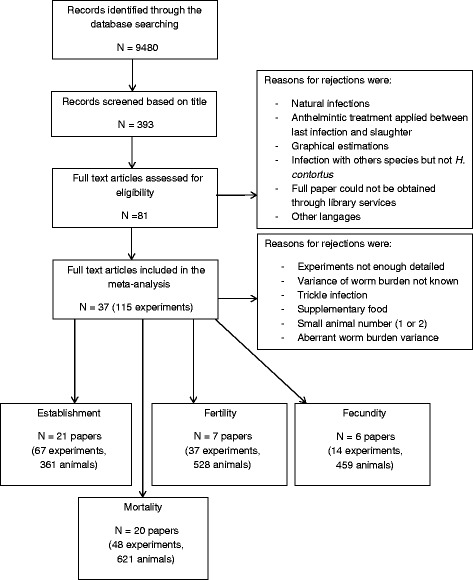
Diagram of the paper selection from the database Web of Science and associated eligibility criteria

Figure 1 provides a description of the paper selection steps and the associated selection criteria. A total of 9480 references were retrieved from Web of Sciences that were narrowed down to 383 following title-based selection. The first review phase of the papers led to a selection of 81 eligible publications, reduced with the second review phase to 37 papers that estimate at least one *H. contortus* life history trait [21, 32–67]. This final database (see Additional file 2) comprised a total of 982 animals used in 115 different experiments (using different experimental conditions as the duration of infection and/or the infection dose).

Table 2 summarizes the details and available life history traits for each paper kept in the final database and the distribution of the experiment number over the different explanatory factors is shown in Additional file 3: Table S1.

**Table 2:** Characteristics of the studies included in the final database to estimate at least one of the key life history traits of *Haemonchus contortus*

Reference	Country	No. of experiments	No. of animals	Breed	Age (months)	Immune past	Duration of infection (days)	Infection dose	No. of experiments			
									E	μ	F	f
[32]	Australia	6	22	Merino		Naïve: 20,000 L_3_	7–21–24–84	5000–10,000	5	1		
[33]	Australia	5	21	Merino	5	Naïve-Natural	7–21–24	5000–20,000	5			
[34]	Australia	5	22	Merino	2.5–6.5	Natural: 5000 L_3_–6000 L_3_	9–12–30–58	5000–6000–10,000	4	1		
[35]	Australia	4	15	Merino		Naïve	21	10,000	4			
[36]	Australia	2	12	Merino	8.5–36	Naïve: 20,000 L_3_	21	10,000	2			
[37]	Australia	2	10	Merino	9.25–14	Naïve	24–28	5000–10,000	2			
[38]	Australia	2	9	Merino		Naïve-Natural	21–26	5000–10,000	2			
[39]	Iraq	2	10	Awassi-Merino	5.5	Natural	39	10,600–13,300		2		
[40]	Brazil	1	6	Santa Ines	3	Natural	40	4000		1		
[41]	Mexico	1	4	Suffolk × Dorset	6.5	Naïve	42	10,000		1		
[42]	Brazil	2	20	Corriedale-Crioula Lanada	3	Naïve	84	3560–4900		2		
[43]	UK	1	5	Blueface Leicester × Scottish blackface	4.5	Naïve	35	5000		1		
[44]	Brazil	1	7	Suffolk	3	Natural	28	4000	1			1
[45]	Scotland	1	3	Scottish Blackface (3/4)	8	Natural	27	5000	1			
[46]	France	4	20	Barbados Black Belly-Romane	12	20,000 L_3_	60	10,000		4		
[47]	USA	23	69	Dorset	6.5	Naïve: 30,000	28–49–70	30,000	7	16	23	
[48]	Mexico	1	6	Colombian breed	7	Naïve	51	5000		1		
[49]	USA	1	9	Dorset	8	10,000 L_3_	35	10,000		1		
[50]	Mexico	1	5	Pelibuey × Dorper	3	Naïve	41	3000		1	1	1
[51]	Australia	4	22	Merino (genetically resistant) Merino	5.5	Naïve: 20,000	28–42	20,000	2	2		
[52]	Australia	1	3	Merino	12	Natural	28	20,000	1			
[53]	Spain	2	17	Canaria-Canaria Hair Breed	8	Natural	28	20,000	2			2
[54]	UK	1	4	Dorset	3	Naïve	22	5000	1			
[55]	Germany	1	100	German Merino and 4 crossbreds	3	Naïve	49	5000		1	1	
[56]	Hungary	3	70	Hungarian Merino	3	Naïve: 7000 L_3_	48–85	7000		3		
[57]	Ethiopia	2	12	Ethiopian highland sheep	11	Natural	91	4000–6000		2		
[58]	France	2	10	Préalpes Ile de France	2.5	Naïve	73–87	10,000–50,000		2		
[59]	USA	2	24	St Croix-Dorset × Rambouillet × Finnsheep	4.5	12,000	27	10,000	2		2	
[60]	South Africa	1	3	Mutton Merino	6	Naïve	42	50,000		1		
[61]	France	4	268	Romane × Barbados BlackBelly BC	3	Natural	42	5,000		4	4	
[62]	France	2	44	Romane × Barbados BlackBelly BC	5	Naïve	30	10,000	2		2	2
[21]	The Netherlands	4	18	Texel	8	Naïve: 5000–10,000–40,000	28	5000	4		4	
[63]	France	8	38	Barbados Black Belly-INRA 401	6	Naïve: 10,000 L_3_	4–30	10,000	8			4
[64]	France	2	8	Barbados Black Belly-INRA 401	8	Naïve	16	10,000	2			
[65]	New Zealand	2	26	Romney-Romney selected for greasy fleece	14.5	Natural	28	4000	2			
[66]	Ethiopia	1	6	Indigenous breed	1	Naïve	84	5000		1		
[67]	USA	8	34	St Croix-Florida Native-Dorset × Rambouillet	9.5	Naïve: 20,000	7–14	16,000–20,000	8			

In addition, the proportion of female worms was estimated from 28 experiments that indicated female and male worm numbers separately. The mean (± standard error, SE) proportion of female worms was 0.51 ± 0.006, which corresponds to a female: male sex ratio of 1.04:1. Estimates for other life history traits of the parasitic phase of *H. contortus* are provided in Table 3.

**Table 3:** The results of the final linear models to estimate each life history trait of the parasitic phase of *H. contortus*

Life history traits	Explanatory factors	Estimation of means	*SE*	*P*
Larval establishment rate (proportion of ingested larvae that develop into immature or adult worms)				
Infection duration				0.04
	4–9 dpi	0.09	0.08	
	12–16 dpi	0.27	0.05	
	21–30 dpi	0.23	0.03	
Infection dose				0.004
	4000–5000 L_3_	0.25	0.05	
	10,000 L_3_	0.22	0.03	
	16,000–20,000 L_3_	0.05	0.07	
	30,000 L_3_	0.26	0.08	
Host age				0.007
	2.5–5 months	0.29	0.06	
	5.5–10 months	0.21	0.02	
	12–36 months	0.08	0.07	
Adult mortality rate (Proportion of adult worms that die per day)				
Host GIN resistance status				0.003
	Susceptible	0.039	0.009	
	Resistant	0.065	0.011	
	Unknwon	0.048	0.009	
Isolate status				0.005
	Susceptible to anthelmintics or non-adapted to sheep resistance isolate of *H. contortus*	0.044	0.009	
	Isolate adapted to sheep resistance^a^	0.069	0.033	
	Isolate resistant to anthelmintics	0.062	0.012	
	Unknown	0.026	0.003	
Host age				0.005
	1.2–3 months	0.045	0.009	
	4.5–8 months	0.059	0.010	
	11–12 months	0.047	0.009	
Previous host exposure				< 0.001
	Immunologically naive	0.049	0.009	
	Suspected natural pre-infection	0.039	0.010	
	Experimental abbreviated pre-infection	0.063	0.009	
Female fertility (number of eggs/female/day)				
Host GIN resistance status				< 0.001
	Susceptible	4545.2	310.0	
	Resistant	2740.9	361.5	
	Unknown	3465.6	513.1	
Infection duration				< 0.001
	27–30 dpi	2136.5	308.9	
	41–50 dpi	2270.6	204.3	
	70 dpi	6344.7	821.2	
Previous exposure of host				< 0.001
	Immunologically naive host	4626.8	372.0	
	Experimental pre-infection	2541.0	377.8	
Population level fecundity (number of eggs/gram of faeces)^b^				
Host GIN resistance status				< 0.001
	Susceptible	13536.8	1429.1	
	Resistant	4837.4	918.8	
	Unknown	9563.8	992.7	
Infection duration				0.002
	27–28 dpi	5032.6	1034.9	
	30 dpi	18032.4	2575.9	
	41–49 dpi	4873.0	415.6	

^a^By serial passages in resistant host

^b^The host age was not included in the models for this trait due to a limited dataset

*Abbreviation*: *SE* standard error

### Larval establishment rate

Average larval establishment rate was 0.24 ± 0.02. The final model, including all the significant explanatory factors, explained 49.5% of total variance (Table 3).

**Fig. 2:**
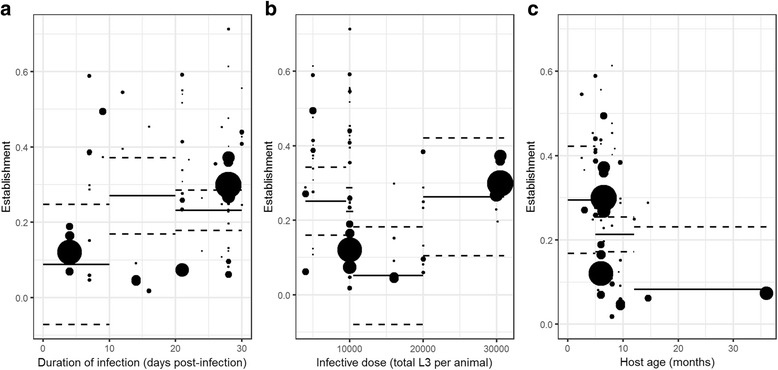
Estimations of the larval establishment according to the slaughter date after infection **a**, the infection dose **b** and the host age **c**. Point size represents the weight associated to each experiment. The solid black lines represent the estimations in the final models and their associated confidence intervals (dashed lines)

The *H. contortus* establishment rate peaked between 10 and 19 dpi (Fig. 2a). Larval establishment rate significantly decreased with the size of the infection dose from 10,000 to 20,000 L_3_ (Fig. 2b), but was highest for the greatest infection dose (result not significant). A reduced proportion of larvae reached adult stages in older hosts (Fig. 2c). This result was consistently obtained, with or without experiments using older sheep than usual age, i.e. 36-month-old *vs* 7 months on average for remaining studies (results not shown).

In our meta-analysis, the proportion of immature stages significantly decreased after 20 dpi and almost disappeared after 40 dpi (Additional file 4: Figure S1), except within a few underpowered experiments from the same paper [47] performed at 49 and 70 dpi.

### Adult mortality rate

**Fig. 3:**
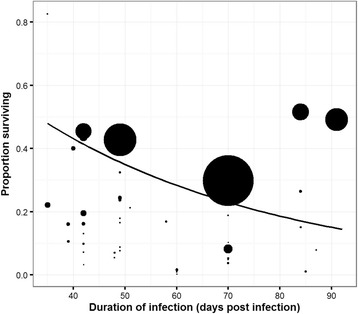
Proportion of surviving worms (i.e. worm burden/infection dose) according to the time post-infection. Point size represents the weight associated to each experiment. The line is based on the average of the adult mortality rate (μ) with the adult mortality equation (*exp*(−*μt*), where *t* is the number of days post-infection)

**Fig. 4:**
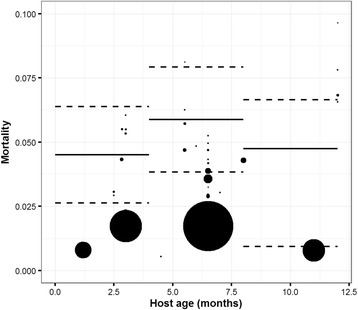
Estimations of the adult mortality according the host age. Point size represents the weight associated to each experiment. The solid black lines represent the estimations in the final models and their associated confidence intervals (dashed lines)

The average (± SE) daily *per capita* adult mortality rate was 0.021 ± 0.002, resulting in a mean life expectancy (1/mortality rate) of 50 days. The proportion of worms surviving (worm burden/infection dose) with time was plotted for every experiment (Fig. 3). As expected, an exponential decrease of the survival probability was observed. The explanatory factors selected as significant in the final model (Table 3) explained 64.6% of the heterogeneity in adult mortality. Strong discrepancies in study’s contribution were observed (Fig. 4).

Significant variations in adult mortality rates were observed according to the *H. contortus* isolate used in the experiment. Susceptible isolates had a significantly higher adult mortality rate than isolates with an unknown resistance. Although not significant, anthelmintic resistant isolates (thiabendazole-resistant strain) demonstrated higher estimated adult mortality rates, and isolates resistant to the immune response had the highest adult mortality rate estimate.

Other explanatory factors were host resistance (*F*_(2,37)_ = 6.82, *P* = 0.003), previous exposure of the host to *H. contortus* (*F*_(2,37)_ = 11.385, *P* < 0.001) and host age (*F*_(2,37)_ = 6.039, *P* = 0.005). Resistant sheep and sheep previously infected with *H. contortus* experimentally eliminated worm burdens faster than their susceptible and immunologically naive counterparts. Adult mortality rates increased with host age (except for individuals aged from 8 to 12 months old, result not significant). In this class, two experiments were high-weighted and decreased the adult mortality rate estimation by erasing the trend of the other experiments.

### Female fertility and fecundity

**Fig. 5:**
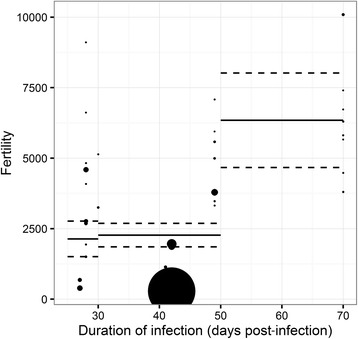
Estimations of the female worm fertility according the slaughter date after infection. Point size represents the weight associated to each experiment. The solid black lines represent the estimations in the final models and their associated confidence intervals (dashed lines)

The average (± SE) female fertility was 1295.9 ± 280.4 eggs per female worm per day. It was strongly associated with the duration of infection (*F*_(2,31)_ = 12.343, *P* < 0.001): female fertility increased with the duration of infection (Fig. 5). Female fertility was less in previously challenged hosts and in resistant breeds compared to their immunologically naive counterparts and susceptible breeds. The explanatory factors considered in the model explained 85.11% of the heterogeneity.

The average faecal egg count at necropsy was 908.5 ± 487.1 eggs per gram of faeces. This fecundity was also affected by the host resistance status (*F*_(2,9)_ = 72.341, *P* < 0.001) and by the duration of infection (*F*_(2,9)_ = 13.171, *P* = 0.002). Fecundity strongly decreased from 30 dpi (Additional file 5: Figure S2) whereas the female fertility continued to increase until 70 dpi (Fig. 5).

### Phenotypic correlations between life history traits

As shown on Table 4, the adult mortality rate was negatively correlated with female fertility (*r*_(22)_ = -0.49, *P* < 0.05). Sex ratio was significantly correlated with all other life history traits: negatively correlated with both larval establishment rate (*r*_(12)_ = -0.77, *P* < 0.01) and female worm fertility (*r*_(5)_ = -0.9, *P* < 0.05) and positively correlated with adult mortality rate (*r*_(12)_ = 0.69, *P* < 0.05).

**Table 4:** Pearson’s correlation coefficients between the life history traits of the parasitic phase of *H. contortus* (weighted by the number of animals in the experiment)

	Larval establishment rate (E)	Adult mortality rate (*μ*)	Female fertility (F)	Population level fecundity (f)	Sex ratio
E		ne	-0.07	0.39	-0.77**
*μ*			-0.49*	-0.88*	0.69*
F				0.58*	-0.9*
f					-0.86
Sex ratio					

*P < 0.05; **P < 0.01

*Abbreviation*: *ne* No estimate (less than two experiments)

## Discussion

To our knowledge, this meta-analysis is the first to collect and summarize the available information on the main life history traits of the parasitic phase of *H. contortus*. The quantification of each parameter, associated with a variation, provides a good foundation to calibrate future epidemiological models describing the parasitic phase of *H. contortus*.

To ensure the quality of our estimates, several criteria were integrated. First, we selected papers giving arithmetic means and variance for two traits: worm burden and faecal egg counts. For the few papers, which gave individual measurements for these two traits, aggregated values were calculated (arithmetic mean and variance). This conservative strategy allowed for increased information from a greater number of studies to be included. This is in contrast to a similar meta-analysis carried out for *T. circumcincta,* which only considered individual worm burden measurements [18]. Like many parametric tests, the analysis of variance assumed the data were normally distributed. However, the traits studied here were non-normal and presented a skewed distribution. Fortunately, the ANOVA test is not very sensitive to moderate deviations from normality; simulation studies, using a variety of non-normal distributions have shown that the false positive rate is not much affected by not adhering to this assumption [68]. In our case, a log-transformation was applied to normalize the life history traits; the results were the same with and without this transformation confirming the robustness of ANOVA. Secondly, using the inverse variance of each life history trait to weight the mean estimate is the most robust way to analyse data reported as a mean with measurement standard deviation and animal number [69]. Studies which only reported means (without the associated measure of variance) or graphical values had to be excluded from further analysis.

Larval establishment and adult mortality are two interwoven traits. Indeed, while most larval stages will evolve into mature adults within 15 days [70], delays can occur that result in larval recovery for up to 30 days after infection [63]. To provide the best larval establishment rate estimates, i.e. the total proportion of infective larvae reaching the adult stage, worm count data up to 30 dpi were considered. Usually, *H. contortus* egg output increases between this stage of infection and 30 dpi as seen in longitudinal faecal egg count sampling (Additional file 5: Figure S2), suggesting that either female worm fertility increases, or that the total number of mature adult stages increases, or both. Therefore, we considered that adult mortality between 15 and 30 dpi was negligible and not to be taken into account. To calculate the number of female worms, a sex ratio of 0.5 was assumed in the material and methods and the present meta-analysis corroborates this sex ratio. This value is a slightly lower than estimations from another meta-analysis which considered several nematode species simultaneously [71].

The success of the nematode in terms of fitness, based on the ability to establish and to produce offspring, relies on both parasite density-dependent constraints and host immune response [72]. Density-dependent stunting of female fertility has been reported for various parasitic species like *H. contortus* in sheep [73], O*. ostertagi* in cattle [16], *Trichostrongylus retortaeformis* in rabbits [72], *Strongyloides robustus* in squirrels [74] or *Syngamus trachea* in crows [75]. From our data, the infection dose did not significantly impact *H. contortus* female fertility, nor its adult mortality rate, but it affected the larval establishment rate. This unexpected result may be explained by the homogenous and relatively small infection doses used in our database to estimate female fertility and adult mortality which were mainly between 5000 L_3_ and 10,000 L_3_ and thus lower than the 10,000 L_3_ threshold where a density dependence effect is generally expected [73]. The only experiments with an infection dose of 30,000 L_3_ used a small sample size (three animals) leading to highly variable estimations. In addition, all these experiments came from the same paper ([47]) with specific environmental conditions (i.e. parasite strain, sheep breed, body weight, sheep sex). In natural infections, the cumulative level of infection is probably lower than 10,000 L_3_ larvae (J. Cabaret, personal communication). The results gathered in our study have confirmed the negative impact of the host immune status on both *H. contortus* fertility and mortality, as reported for other trichostrongylid species [76]. These findings tend to favor a negative effect of steric crowding on larval establishment. It remains unresolved whether the observed effects on *H. contortus* mortality and fertility were due to direct host responses, or from density-dependent effects mediated by the host immune response as demonstrated in *Strongyloides ratti* infections [77]. Further, the infection dose is only an indirect approximation of the actual established worm burden so that density dependence may be overlooked.

The negative impact of host genetic resistance on *H. contortus* mortality rate and female fertility corroborates previous results comparing resistant and susceptible breeds [78, 79] or lines of sheep with divergent resistance status [62, 80]. Such an observation also links with the proposed framework for the immune response against *T. circumcincta* [81].

Surprisingly, naturally pre-challenged sheep seemed to exert a significantly lighter response on adult mortality rate than their experimentally pre-challenged or naïve counterparts. This may be a result of the misclassification of studies due to a lack of information in the material and methods section. For example, some experimental animals were explicitly mentioned as naturally infected (even if pasture infectivity was not stated) and treated to remove presence of parasites, but some others were mentioned as receiving preventative treatments but not really faced parasite challenge.

*Haemonchus contortus* is well known as one of the most prolific sheep parasite [82]. Our estimates indicate a high female fertility of 1295.9 ± 280.4 eggs/female/day on average, which outperforms other trichostrongylids [82]. It is usually postulated that this high female fertility will result in a huge population size showing a high degree of genetic variability [83]. However, our estimates also suggest that larval establishment constitutes a strong bottleneck as only a quarter of the ingested larvae will have an opportunity to mate and pass on their genetic material to subsequent generations. This may constrain the expected population size of *H. contortus*.

The mean estimated life expectancy of 50 days by this meta-analysis underestimated the predicted half-life of an established worm burden of 69 days as described by Barger & Le Jambre [84]. Indeed, the equation used to estimate the adult mortality rate [47] did not take into account the establishment of the ingested larvae (assumed as 100%) leading to an overestimation of the adult mortality rate. The analyses were also made with the equation of Barger & Le Jambre [84] using the average larval establishment rate estimated by our meta-analysis. Unfortunately, for a third of the experiments this estimate is higher than the effective larval establishment rate of the experiment, leading to a negative adult mortality rate (replaced by 0 in this case) and thus biasing the adult mortality estimates.

Interestingly, the more female-biased the *H. contortus* population was, the lower the establishment and fertility rates were, yet with higher adult mortality rates. These correlations are difficult to interpret as the higher proportion of females could be a cause, or a consequence, of the two other traits. Female worms are thought to live longer than males, which could explain why populations exhibiting high adult mortality rate also show more females than males [71]. The higher proportion of females in low larval establishment rates may be a result of a better female survival capacity. The decrease in female fertility observed for female-biased populations may be due to either a competition for males, or competition for resources as their nutrient requirements are usually higher than for males [71].

## Conclusions

To our knowledge, this comprehensive systematic review of the parasitic phase of *H. contortus* is the first to summarize these extensive data and to provide average overall life history trait estimates. These informative estimations are very helpful to parameterize epidemiological transmission models more accurately. This meta-analysis also gives evidence for density dependence of larval establishment, for host age affecting larval establishment and adult mortality and shows that an improved immune response, induced by the resistance status of the breed or by a previous exposure to the parasites, affects adult mortality and female fertility. An improved understanding of the parasitic life-cycle will allow us to evaluate the impact of different control strategies on parasitic infection with an increased confidence in the output predictions of dynamic models. For example, the sustainability of treatments and genetic selection strategies could be evaluated both on the infection level of the flock and on the increase of resistant alleles in parasite strains. The impact of environmental factors on free living stages also lacks precise estimates and a meta-analysis of these would certainly help to conclude on this point.

## Additional files

Additional file 1: Text: Calculation method to estimate the variance of each life history trait. (DOCX 30 kb)

Additional file 2. Data: Final database of all the papers included in the meta-analysis after all the selection criteria. (XLSX 33.4 kb)

Additional file 3: Table S1. Distribution of the experiment number, of the animal number and of the experiment weight over different levels of the explanatory factors. (DOCX 17 kb)

Additional file 4: Figure S1. Proportion of immature stages on the total worm burden according to the infection duration post infection. Point size represents the weight associated to each experiment. The solid black line represents the fitting by polynomial model (degree 2) and the dashed lines represent its confidence interval. (TIFF 2774 kb)

Additional file 5: Figure S2. Faecal egg count according to the infection duration post infection. Black cross sizes represent the weight associated to each faecal egg count reported. Grey crosses represent faecal egg count reported without the associated measure of variance. (TIFF 2600 kb)

## Abbreviations

DFP: Daily faeces production
FEC: Faecal egg count
*FEC*_*n*_: Faecal egg count at necropsy
*F*_*p*_: Proportion of female worms
ID: Infection dose
SE: Standard error
WB: Worm Burden.
