# Supplementary material for: Meta-analysis of the parasitic phase traits of Haemonchus contortus infection in sheep
Source: Parasit Vectors. 2017 Apr 24;10:201. doi: 10.1186/s13071-017-2131-7 (PMC5402645; doi:10.1186/s13071-017-2131-7)
Supplement: Supplementary file 1 — Text: Calculation method to estimate the variance of each life history trait. (DOCX 30 kb) [file 13071_2017_2131_MOESM1_ESM.docx]

**Additional file 1**: Calculation method to estimate the variance of each life history trait. These calculations were modified from equations used by Verschave [1,2].

For each experiment *j*, the average worm burden ${WB}_{j}=\frac{1}{n_{j}}\sum_{i=1}^{n_{j}} x_{ij}$ is assumed to be normally distributed:

$${WB}_{j}\mathcal{\sim N(}\mu_{x},\frac{\sigma_{x}^{2}}{n_{j}})$$

where $x_{ij}$is the worm burden of the animal *i* in the experiment *j* and $n_{j}$ the number of animal in the experiment *j*.

In the same idea, the fecundity is noted${FEC}_{j}=\frac{1}{n_{j}}\sum_{i=1}^{n_{j}} y_{ij}$, where $y_{ij}$ is the fecundity of the worm population in the animal *i* in the experiment *j.*

1. Establishment trait

The establishment rate (ε) is commonly estimated by the proportion of worms retrieved at necropsy (L_4_, immature stages and adult worms) divided by the number of larvae inoculated (worm burden/infective dose = *WB/ID*). So:

$$V\left( \varepsilon\right)=V\left( \frac{WB}{ID} \right)=\frac{1}{{ID}^{2}}V\left( WB \right)=\frac{{S.E.(WB)}^{2}}{{ID}^{2}}$$

where $S.E.\left( WB \right)=sd\left( WB \right)/\sqrt{n}$

1. Mortality trait

The mortality rate (µ) was estimated as $\mu=-\frac{ln\left( \frac{WB}{ID} \right)}{t}$ from Coyne and Smith [3] and its variance was calculated as follows:

$$V\left( \mu\right)=V\left( -\frac{ln\left( \frac{WB}{ID} \right)}{t} \right)=\frac{1}{t^{2}}V\left( ln\left( \frac{WB}{ID} \right) \right)=\frac{1}{t^{2}}V\left( ln\left( Z \right) \right)$$

where $Z=\frac{WB}{ID}$

Propriety: Using the Taylor series, supposing X as a random variable, and *g(.)* a transformation :

$$V\left( g\left( X \right) \right)\approx\left( g^{'}\left( E\left( X \right) \right) \right)^{2}V\left( X \right)$$

If $g\left( . \right)=ln()$, then $V\left( ln\left( X \right) \right)\approx\frac{\sigma_{X}^{2}}{\mu_{X}^{2}}$

So,

$$V\left( ln(Z) \right)=\frac{\sigma_{Z}^{2}}{\mu_{Z}^{2}}=\frac{\frac{{S.E.(WB)}^{2}}{{ID}^{2}}}{\frac{WB^{2}}{ID^{2}}}=\frac{S.E.(WB)^{2}}{WB^{2}}$$

and,

$$V\left( \mu\right)=\frac{1}{t^{2}}V\left( ln\left( Z \right) \right)=\frac{{S.E.\left( WB \right)}^{2}}{\left( t*WB \right)^{2}}$$

1. Fertility trait

The fertility is defined as the average number of eggs excreted by a female worm per day. It is estimated as:

$$\frac{{FEC}_{n}*DFP}{WB*F_{p}}$$

where ${FEC}_{n}$is the faecal egg count at necropsy; *DFP* the daily faeces production and $F_{p}$ is the proportion of females.

Propriety: Using Taylor expansions and Stuart and Ord [4], consider two random variables X and Y, we have:

$$V\left( \frac{X}{Y} \right)\approx\frac{1}{E(Y)^{2}}V\left( X \right)+\frac{E\left( X \right)^{2}}{E\left( Y \right)^{4}}V\left( Y \right)-2\frac{E(X)}{E\left( Y \right)^{3}}Cov(X,Y)$$

The variance is approximately equal to:

$$V\left( \frac{{FEC}_{n}*DFP}{WB*F_{p}} \right)\approx\frac{1}{\left( F_{p}*E\left( WB \right) \right)^{2}}V\left( {DFP*FEC}_{n} \right)+\frac{\left[ E\left( DFP*{FEC}_{n} \right) \right]^{2}}{\left[ E\left( WB*F_{p} \right) \right]^{4}}V\left( WB*F_{p} \right)$$

$$-2*\frac{E\left( DFP*{FEC}_{n} \right)}{\left[ E\left( WB*F_{p} \right) \right]^{3}}cov\left( DFP*{FEC}_{n},WB*F_{p} \right)$$

Assuming *WB* and ${FEC}_{n}$ independent variables,

$$V\left( \frac{{FEC}_{n}*DFP}{WB*F_{p}} \right)=\frac{1}{\left( F_{p}*WB \right)^{2}}{DFP}^{2}V\left( {FEC}_{n} \right)+\frac{{DFP}^{2}*{{FEC}_{n}}^{2}}{{F_{p}}^{4}{WB}^{4}}{F_{p}}^{2}V\left( WB \right)=\frac{{DFP}^{2}*{{FEC}_{n}}^{2}}{{F_{p}}^{2}{WB}^{2}}\left[ \frac{{S.E.({FEC}_{n})}^{2}}{{{FEC}_{n}}^{2}}+\frac{{S.E.(WB)}^{2}}{{WB}^{2}} \right]$$

1. Sex ratio trait

The sex ratio is defined as the proportion of female worms in the total number of adult worms ($N_{fem}/AW)$. As for the fertility rate trait, the variance is estimated by the ratio of two random variables:

$$V\left( Sex ratio \right)=\frac{1}{\left[ E\left( AW \right) \right]^{2}}V\left( N_{fem} \right)+\frac{\left[ E\left( N_{fem} \right) \right]^{2}}{\left[ E\left( AW \right) \right]^{4}}V\left( AW \right)-2\frac{E\left( N_{fem} \right)}{\left[ E\left( AW \right) \right]^{3}}cov\left( AW,N_{fem} \right)=\frac{{N_{fem}}^{2}}{{AW}^{2}}\left[ \frac{V\left( N_{fem} \right)}{{N_{fem}}^{2}}+\frac{V(AW)}{{AW}^{2}} \right]$$

REFERENCES

1. Verschave SH, Vercruysse J, Claerebout E, Rose H, Morgan ER, Charlier J. The parasitic phase of Ostertagia ostertagi: quantification of the main life history traits through systematic review and meta-analysis. Int J Parasitol. 2014;44:1091–104.

2. Verschave SH, Rose H, Morgan ER, Claerebout E, Vercruysse J, Charlier J. Modelling Cooperia oncophora: Quantification of key parameters in the parasitic phase. Vet Parasitol. 2016;223:111–4.

3. Coyne MJ, Smith G. The mortality and fecundity of *Haemonchus contortus* in parasite-naive and parasite-exposed sheep following single experimental infections. International Journal for Parasitology. 1992;22:315–25.

4. Stuart A, Ord J. Kendall’s Advanced Theory of Statistics, Vol. 1: Distribution Theory. 6th ed. New York: Oxford University Press; 1998.
