# Supplementary material for: Meta-analysis of the parasitic phase traits of Haemonchus contortus infection in sheep
Source: Parasit Vectors. 2017 Apr 24;10:201. doi: 10.1186/s13071-017-2131-7 (PMC5402645; doi:10.1186/s13071-017-2131-7)
Supplement: Supplementary file 3 — Distribution of the experiment number, of the animal number and of the experiment weight over different levels of the explanatory factors. (DOCX 17 kb) [file 13071_2017_2131_MOESM3_ESM.docx]

**Additional file 2. Table S1** Distribution of the experiment number, of the animal number and of the experiment weight over different levels of the explanatory factors.

|  | | **Establishment**  (proportion of ingested larvae that develops into immature or adult worms) | **Mortality**  (Proportion of adult worms that die per day) | **Fertility**  (Number of eggs produced by an adult female per day) | **Fecundity**  (Number of eggs / gram of faeces*)* |
| --- | --- | --- | --- | --- | --- |
| **Host resistance** | |  |  |  |  |
|  | Resistant | **14**  (*96* – 16730.48) | **6**  (*157* - 458225) | **4**  (*176* -0.000235) | **4**  (*176* – 1.63e-04) |
|  | Susceptible | **47**  (*232* – 45400.5) | **30**  (*246* -7068977) | **26**  (*217*-0.000090) | **3**  (*148* – 2.25e-06) |
|  | Unknown | **6**  (*33* – 650.5646) | **12**  (*218* - 5030974) | **7**  (*135*- 0.000030) | **7**  (*135* – 1.38e-05) |
| **Mean age in months (min-max)** | | 2.5 - 36 | 1.2 - 12 | 2.8 - 8 | 2.8- 8 |
|  | Class 1 | [2.5 - 5]  **13**  (*108* -9056.318) | [1.2 - 3]  **16**  (*489* – 5191526) | [2.8 - 3]  **6**  (*373* – 0.000284) |  |
|  | Class 2 | [5.5 – 10]  **38**  (*169* - 43567.88) | [4.5 - 8]  **25**  (*97* – 5616842) | [4.5 - 6]  **4**  (*68* – 0.000023) |  |
|  | Class 3 | [12 - 36]  **5**  (*41* – 6257.906) | [11 - 12]  **6**  (*32* – 1711172) | [6.5 - 8]  **27**  (*87* – 0.000048) |  |
|  | Unknown | **11**  (*43* – 3899.452) | **1**  (*3* – 38636.02) | **0** |  |
| **Isolate status** | |  |  |  |  |
|  | Isolate adapted to sheep resistance ^(a)^ | **1**  (*7* –115.3721) | **2**  (*10* – 18292.91) | **0** |  |
|  | Isolate resistant to anthelmintics | **1**  (*4* – 1537.87) | **3**  (*70* – 144471.8) | **0** |  |
|  | Susceptible to anthelmintics or non-adapted to sheep resistance isolate of *H. contortus* ^(b)^ | **5**  (*22* – 358.1386) | **5**  (*35* – 287838.3) | **4**  (*18* – 0.000002) | **4**  (*18* – 1.38e-06) |
|  | Unknown | **60**  (*328*– 60770.18) | **38**  (*506*- 12107573) | **33**  (*510* – 0.000353) | **10**  (*441* – 1.77e-04) |
| **Previous exposure to *H. contortus*** | |  |  |  |  |
|  | Naïve | **317**  (*150*– 32621.1) | **23**  (*209* -6810693) | **17**  (*188* – 0.000055) | **5**  (*152* – 1.31e-05) |
|  | Natural infection | **13**  (*95* – 10377.22) | **5**  (*52* -2794323) | **0** | **0** |
|  | Experimental infection | **23**  (*116*– 19783.24) | **20**  (*360* - 2953159) | **20**  (*340* – 0.000300) | **9**  (*307* – 1.66e-04) |
| **Number of days post-infection (min-max)** | | 4 – 30 | 35 – 91 | 27-70 | 27-49 |
|  | Class 1 | [4 - 9]  **4**  (*18* – 12556.25) | [35 - 50]  **25**  (*495* – 5268581) | [27 - 30]  **15**  (*107* – 0.000055) | [27 – 28]  **6**  (*42* – 3.2e-06) |
|  | Class 2 | [12 - 16]  **17**  (*71* – 14121.66) | [from 51 - 73]  **15**  (*59* – 4453469) | [41 - 50]  **14**  (*397* – 0.000293) | [30]  **2**  (*44* – 4.47e-07) |
|  | Class 3 | [21 - 30]  **46**  (*272*– 36103.65) | [84 - 91]  **8**  (*67* – 2836126) | [70]  **8**  (*24* – 0.000008) | [41 - 49]  **6**  (*373* – 1.75e-04) |
| **Infection doses in L_3_ (min-max)** | | 4000 - 30488 | 3000 - 50000 | 3000 – 30488 | 3000 - 10000 |
|  | Class 1 | [4,000 - 50,000]  **18**  (*95* – 9424.054) | [3,000 - 5,000]  **9**  (*154* – 3564562) | [3,000 - 5,000 ]  **6**  (*123* -0.000046) | [3,000 – 5,000]  **6**  (*123* – 1.34e-05) |
|  | Class 2 | [10,000]  **29**  (*181* – 26706.9) | [6,000 - 20,000]  **17**  (*391* – 4113650) | [10,000]  **8**  (*336* -0.000286) | [10,000]  **8**  (*336* – 1.65e-04) |
|  | Class 3 | [16,000 - 20,000]  **13**  (*64* – 10032.32) | [29,931 - 50,000]  **22**  (*76* – 4879964) | [29,931 - 30,488]  **23**  (*69* -0.000023) |  |
|  | Class 4 | [29,931 - 30,488]  **7**  (*21* – 16618.28) |  |  |  |

**Number of experiments**; *number of animals*; experimental weight (i.e. inverse variance of each life trait)

^(a)^ by serial passages in resistant host.

^(b)^ isolates specified as susceptible in the material and method of the paper.
