# Supplementary figures and images for: Meta-analysis of the parasitic phase traits of Haemonchus contortus infection in sheep
Source: Parasit Vectors. 2017 Apr 24;10:201. doi: 10.1186/s13071-017-2131-7 (PMC5402645; doi:10.1186/s13071-017-2131-7)

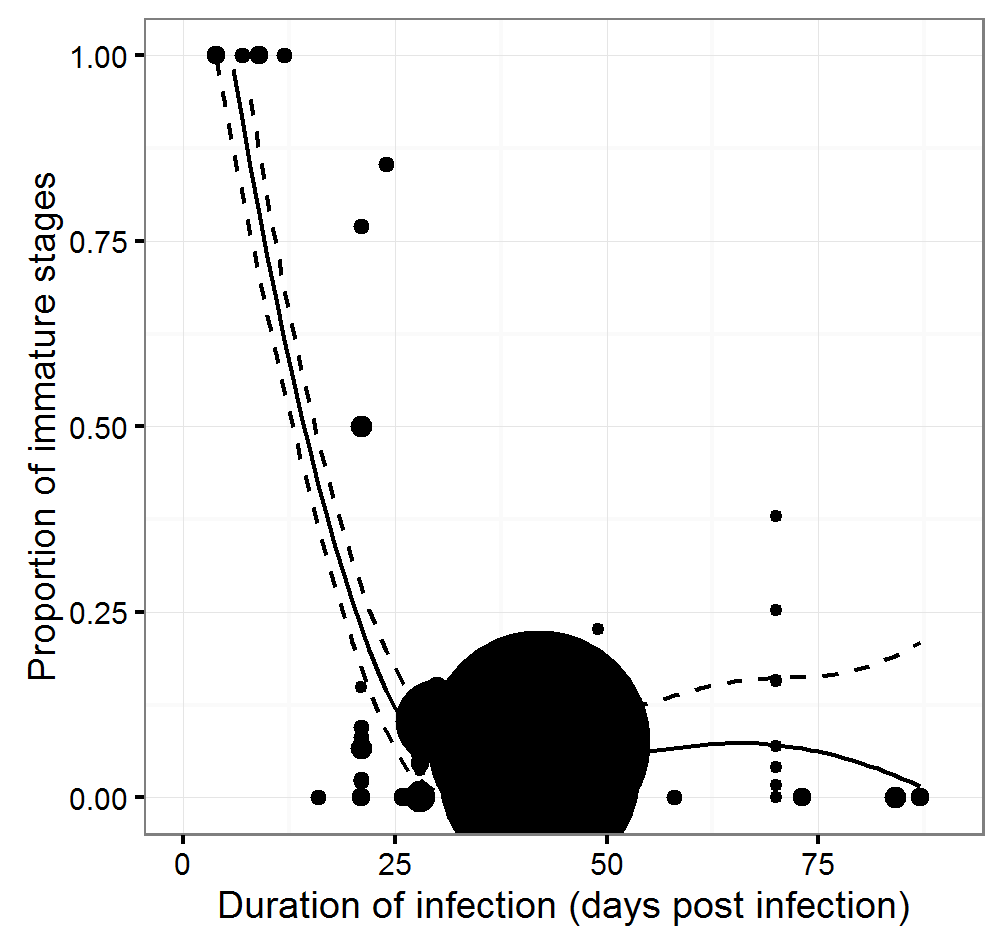

Supplement: Supplementary file 4 — Proportion of immature stages on the total worm burden according to the infection duration post infection. Point size represents the weight associated to each experiment. The solid black line represents the fitting by polynomial model (degree 2) and the dashed lines represent its confidence interval. (TIFF 2774 kb) [file 13071_2017_2131_MOESM4_ESM.tiff]

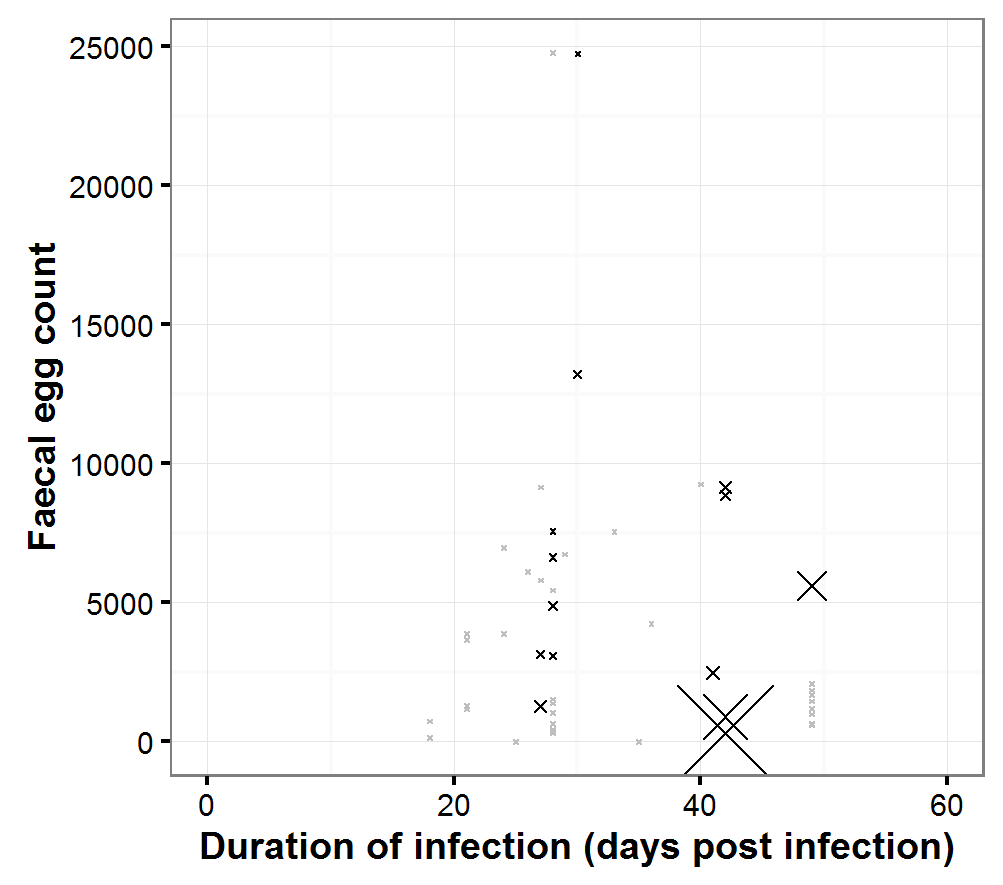

Supplement: Supplementary file 5 — Faecal egg count according to the infection duration post infection. Black cross sizes represent the weight associated to each faecal egg count reported. Grey crosses represent faecal egg count reported without the associated measure of variance. (TIFF 2600 kb) [file 13071_2017_2131_MOESM5_ESM.tiff]
